# Immunoglobulin G (IgG) specific responses to recombinant Qβ displayed MSP3 and UB05 in plasma of asymptomatic *Plasmodium falciparum*-infected children living in two different agro-ecological settings of Cameroon

**DOI:** 10.11604/pamj.2024.47.175.38169

**Published:** 2024-04-09

**Authors:** Loveline Ngu, Herve Ouambo Fotso, Inès Nyebe, Jules Colince Tchadji, Georgia Ambada, Akeleke Ndah, Bloomfield Atechi, Abel Lissom, Philémon Etienne Atabonkeng, George Chukwuma, Vitalis Efezeuh, Park Chae Gyu, Charles Esimone, Jules Clement Assob Nguedia, Eric Achidi Akum, Malachy Okeke, Vincent Pryde Kehdingha Titanji, Wilfred Mbacham, Alain Bopda-Waffo, Godwin Nchinda Wapimewah

**Affiliations:** 1 Laboratory of Vaccinology/Biobanking, Chantal Biya International Reference Center for Research on the Prevention and Management of HIV/AIDS, Yaounde, Cameroon,; 2 Department of Biochemistry, Faculty of Sciences, University of Yaounde I, Yaounde, Cameroon,; 3 Pan African Center for Clinical and Translational Sciences (PANECTS), Yaounde, Cameroon,; 4 Department of Microbiology, Faculty of Sciences, University of Yaounde I, Yaounde, Cameroon,; 5 Department of Animal Biology and Physiology, Faculty Of Sciences, University of Yaounde I, Yaoundé, Cameroon,; 6 Department of Biological Sciences, Faculty of Sciences, University of Bamenda, Bamenda, Cameroon,; 7 Department of Biochemistry, University of Dschang, Dschang, Cameroon,; 8 Department of Medical Laboratory Science, College of Health Sciences, Nnamdi Azikiwe University, Awka, Nigeria,; 9 Department of Biochemistry and Molecular Biology, University of Buea, Buea, Cameroon,; 10 Laboratory of Immunology, Brain Korea 21 PLUS Project for Medical Science, Severance Biomedical Science Institute, Yonsei University College of Medicine, Seoul, Republic of Korea,; 11 Department of Pharmaceutical Microbiology and Biotechnology, Nnamdi Azikiwe University, Awka, Nigeria,; 12 Department of Medical Laboratory Sciences, University of Buea, Buea, Cameroon,; 13 Department of Natural and Environmental Sciences, Biomedical Science Concentration, School of Arts and Sciences, American University of Nigeria, 98 Lamido Zubairu Way, Yola, Nigeria,; 14 Biotechnology Units, Department of Biochemistry and Molecular Biology, University of Buea, Buea, Cameroon,; 15 Biochemistry and Molecular Biology, Indiana University School of Medicine, 635 Barnhill Drive, MS1017Q Lab MS1015, Indianapolis, IN, United States of America

**Keywords:** Asymptomatic *Plasmodium*-infected children, bimodal, monomodal, rainfall, QβUB05, QβMSP3

## Abstract

**Introduction:**

in areas with intense perennial malaria transmission, limited data is available on the impact of environmental conditions especially rainfall on naturally acquired immunity against promising malaria vaccine candidates. For this reason, we have compared IgG antibody responses specific to Plasmodium spp. derived MSP3 and UB05 vaccine candidates, in plasma of children living in two areas of Cameroon differing in rainfall conditions.

**Methods:**

data about children less than 5 years old was collected during the years 2017 and 2018. Next malaria asymptomatic P. falciparum (Pf) infected children were selected following malaria test confirmation. MSP3 and UB05 specific IgG antibody responses were measured in participant´s plasma using enzyme-linked immunosorbent assay (ELISA).

**Results:**

interestingly, IgG antibody responses specific to UB05 were significantly higher (p<0.0001) in Pf-negative children when compared to their asymptomatic Pf-infected counterparts living in monomodal rainfall areas. In contrast, a significantly higher (p<0.0001) IgG response to MSP3 was observed instead in asymptomatic Pf-infected children in the same population. In addition, IgG responses specific to UB05 remained significantly higher in bimodal when compared to monomodal rainfall areas irrespective of children´s Pf infection status (p<0.0055 for Pf-positive and p<0.0001 for negative children). On the contrary, IgG antibody responses specific to MSP3 were significantly higher in bimodal relative to monomodal rainfall areas (P<0.0001) just for Pf-negative children.

**Conclusion:**

thus IgG antibody responses specific to UBO5 are a better correlate of naturally acquired immunity against malaria in Pf-negative Cameroonian children especially in monomodal rainfall areas.

## Introduction

In Cameroon like in most sub-Saharan African countries children because of their fragile immune systems are highly vulnerable to frequent *Plasmodium* species-mediated malaria. Malaria remains a challenging endemic parasitic disease with nearly a million victims yearly, especially within this region where children and pregnant women continue to bear the brunt of the disease. In 2020, for example, Africa alone accounted for 95% of all global cases of malaria and also 96% of all malaria deaths [1]. The malaria pandemic in sub-Saharan Africa is driven by infection with *Plasmodium falciparum* which happens to also be the dominant cause of malaria-mediated mortality in Africa [2].

Repeated exposure of people living in countries endemic to malaria parasite does not lead to complete protective immunity. Instead, people living within these regions developed partial immunity to the disease which left them continuously vulnerable to repeated episodes of malaria [3]. Three different types of naturally acquired immunity have been described for malaria. This includes clinical immunity, anti-parasite immunity, and sterilizing immunity, which is characterized by low-grade asymptomatic parasitaemia [4,5]. Partial naturally acquired immunity (NAI) develops very early in life [5]; a critical component of such immunity being *Plasmodium* species-induced IgG antibody response targeting a number of parasite-derived antigens. Antibodies targeting antigens expressed by merozoites which are blood-stage parasites [6] are known to play vital roles in reducing parasite multiplication thereby preventing infection and clinical disease in long-term residents of endemic areas [7,8]. Antibodies to several asexual blood stage antigens including apical membrane antigen 1 (AMA-1), erythrocyte binding antigen (EBA-175), the merozoite surface proteins (MSPs), reticulocyte-binding protein homologue (Rh5), glutamate-rich protein (GLURP), [8,9] and UB05 [10,11] have been demonstrated to be essential components of naturally acquired malaria-specific immunity. Such immunity is implicated in the reduction of parasite multiplication thereby preventing infection and clinical disease in long-term inhabitants of endemic regions [8-11].

In these regards, high levels of IgG antibody responses and diversity of the target antigens, have been associated with naturally acquired immunity to malaria [8-12]. However, due to inherent polymorphism in the asexual blood-stage antigens, a suitable vaccine candidate for blood-stage parasite must still be determined [12]. Some elements of antibody-mediated immunity to *P. falciparum* have been reported to be strain specific thereby also limiting their utility as global malaria vaccine candidates [9].

In some malaria-endemic countries like Cameroon, there is an uneven distribution of the disease arising from varying intensity of malaria transmission across differing geographical settings [10]. This is mainly driven by differences in rainfall and climatic conditions. Bimodal rainfall areas for example; as a result of differences in rainfall have higher transmission intensity relative to monomodal regions. This implies that children less than five years´ old living in bimodal areas of Cameroon are most likely challenged by a high intensity of malaria parasite transmission. With respect to malaria vaccine candidates, it is not known whether such children will show similar IgG antibody responses to malaria vaccine candidates like those living in monomodal areas. Recently, Kwenti *et al*. demonstrated that IgG and subclass responses against MSP-1_19_ vary considerably in children from the different bioecological strata in Cameroon [12]. To optimize malaria vaccine candidates, it is necessary to select candidates that will give the same outcome across several bio-ecological zones.

The *Plasmodium falciparum* derived UB05 antigen was isolated in the sera of semi-immuned adults living in the Cameroonian rainforest and has been demonstrated as a surrogate marker of naturally acquired protective immunity to malaria [9]. Immunization of albino, a laboratory-bred strain of the house mouse (BALB/c mice) with a recombinant chimera of UB05 and UB09 (UB05-09), blocked not only parasitemia but equally protected from a lethal challenge with *Plasmodium yoelii* 17XL [13]. Similarly, antibodies specific to *Plasmodium falciparum* derived MSP3 have been demonstrated to control *P. falciparum* parasite density by triggering blood monocytes mediated antibody-dependent cellular inhibition (ADCI) [14,15]. In addition, the highly conserved C-terminal end of MSP3 has been assessed in clinical trials with promising outcomes [16].

In this study, we compared the IgG antibody responses specific to previously described malaria vaccine candidates in the plasma of children living in two areas with perennial transmission of malaria but differing in malaria transmission intensity due to climatic conditions. Using recombinant ribonucleic acid (RNA) coliphage Qβ displaying the antigens as previously described in our group antigenicity was determined in the plasma of participants [17]. The recombinant phage QβMSP3 displays the conserved C-terminal 88 AA of the Merozoite Surface Protein 3 (MSP3) [18] whilst QβUB05 bears the previously described malaria antigen UB05 [9].

Cameroon has a wide variety of soils and climates that permit the division of the country into 5 main agroecological zones consisting of a forest zone with monomodal rainfall and a rainforest zone with bimodal rainfall [19]. Whilst previous studies in Cameroon have shown that malaria transmission is perennial in the forested regions [20,21], little is known about the immune response to malaria vaccine candidates in plasma from children from different ecological settings of Cameroon. Therefore, the main objective of this study was to investigate differences in the IgG antibody responses specific to recombinant MSP3 and UB05 in children living in two different agroecological facies with perennial transmission of malaria in Cameroon.

Specifically, IgG antibody responses specific to both antigens (UB05 and MSP3) were compared firstly with respect to *Pf* parasite load; and secondly relative to the rainfall conditions of the study area. This consisted of Buea situated in a forest zone with monomodal rainfall and Bikop deep in a rainforest with bimodal rainfall. Our study is important for selecting malaria vaccine candidates that can give similar immune outcomes across varying malaria transmission intensities thereby providing measurable correlates of immunity. This could enable the design of novel highly efficacious vaccines applicable in all regions irrespective of climatic conditions or transmission intensities.

## Methods

**Description of the study area:** this study was carried out simultaneously in one dense rainforest area with bimodal rainfall (Bikop) compared with another dense rainforest region with monomodal rainfall (Buea). Bikop (3°31'00.0"N 11°25'00.0"E), is a rural health district located in the Center Region of Cameroon, 48 Km away from Yaounde, the City Capital of Cameroon. The Bikop health district covers 28 rural communities with an estimated population of 30,000 inhabitants. This area is moderately accessible in all weathers. The climate is typically equatorial with an average annual temperature of 23.5 °C and mean annual rainfall of 831.7 mm. There are two seasons; the dry season from November to February and June to August; the wet season from March to May and September to November. Agriculture and fishing are the main sources of livelihood [22].

Buea (4°09' 34´´N 9° 14' 12''E), is presently the regional headquarter of the South West Region of Cameroon and is located 318 Km away from Yaounde, the City Capital of Cameroon. The Buea health district covers 67 communities with an estimated population of 200,000 inhabitants, on a surface area of 870 Km^2^ [23,24]. This area is accessible in all weathers. Buea has an equatorial climate with temperature ranges between 20°C to 28°C and annual rainfall ranges between 3000 mm to 5000 mm. There are two major seasons. The rainy season which runs from February to October and the dry season, from November to May. Agricultural, administrative, business, tourism and the University educational sector are the main sources of livelihood in Buea [23].

**Study design:** this was a cross-sectional study involving children under 5 years old. The study was carried out during the months of November 2017, February, and November 2018 in Bikop (bimodal rainfall area) and Buea (monomodal rainfall area). Once ethical clearance and administrative authorization to collect samples were obtained, meetings with the parents were organized, during which the project objectives, methods, and possible benefits/risks were clearly explained. Thereafter, children accompanied by their parents or guardians were invited to participate in the survey and recruited solely on the basis of convenience and accessibility. Children with parental authorization were interviewed in the presence of parents, and using a pretested questionnaire, we collected socio-demographic information and history of previous malaria episodes, and treatment behavior. For each child, a general clinical evaluation was carried. Children with acute symptoms of malaria, fever (>37.5°C) or chills, and children recently sick (three months prior to data collection) or under antimalarial treatment were excluded from the study. Following malaria test confirmation malaria asymptomatic *P. falciparum (Pf)* infected children were recruited for the study. MSP3 and UB05 specific IgG antibody responses were measured in participant´s plasma by ELISA.

### Selection criteria

**Inclusion criteria:** participants were healthy children aged less than 5 years old, attending nursery, or primary schools in the chosen localities. Only children who received parental assent were recruited for the study,

**Exclusion criteria:** excluded from this study were children presenting any condition that might place the participant at undue risk or that can interfere with the study results (e.g. HIV infection, hepatitis B or C, immunosuppressive or immunodeficient condition, febrile state (T>37.5°C), malnutrition, severe chronic disease, corticosteroid therapy). Children with any history of symptomatic malaria or a history of anti-malarial medication in the past three months; and children with a medical condition that would make venipuncture dangerous.

**Data and samples collection:** the sample size was calculated using the standard formula for sample size calculation [25].


$$N=\frac{z^{2}pq}{d^{2}}$$

Where z = the standard normal deviation at 1.96 (which corresponds to a 95% confidence interval), p = the prevalence of asymptomatic malaria in the general Cameroonian population, estimated at 28.9% (1); q = 1 - p; and d = the degree of precision expected = 0.05). During the study period, a total of 123 children in Bikop and 99 children in Buea were enrolled in the study. Based on the selection criteria, only 68 and 66 children in Bikop and Buea respectively, were finally enrolled in the study. Temperature was recorded using an infrared body thermometer. A blood sample was collected using sterile disposable syringes into a 2.5 mL labeled ethylene Diamine Tetra Acetate (EDTA) tubes, at the Bikop Catholic Health Facility and Buea Hospital. While the Rapid Diagnostic Test for malaria was carried out immediately post sample collection on the site after which the remaining blood was transported on ice in a cool box with a temperature between 4 and 10°C to the Chantal Biya International Reference Centre (CIRCB) for serological analyses.

**Ethical considerations:** this study received ethical approval from the Cameroon National Ethics Committee for Human Health Research (Reference numbers 2015/03/561/CE/CNERSH/SP and 2018/01969/CE/CNERSH/SP). All participants provided written informed consent. Data were processed using specific identifiers for privacy and confidentiality purposes. Clinical data generated during the course of this study was provided free of charge to all participants.

### Laboratory investigations

**Malaria diagnosis:** the SD Bioline Malaria antigen uses histidine-rich protein 2 (HRP 2) to detect *P. falciparum* and lactose dehydrogenase (LDH) for *Plasmodium vivax, Plasmodium ovale* and *Plasmodium malariae* [26,27]. This test is a WHO-prequalified test useful for the region where all malaria species are circulated, and it has a sensitivity of 99.7% for *P. falciparum* and 95.5% for *P. vivax*, with a specificity of 99.3% [28]. The tests were used according to the manufacturer´s instructions. To ensure the validity of the results, rapid diagnostic tests (RDTs) were read within a timeframe of a maximum of 15 minutes by two independent laboratory technicians. For microscopic diagnostic of malaria, we used optical microscopy. Optical microscopy of thick and thin stained blood smears remains the standard method for diagnosing malaria. It involves Giemsa staining and examination of malarial parasites [29]. Microscopy was performed as a QC for RDTs negative samples, and also to determine the parasite load.

**Parasite density estimation:** we counted all parasites and white blood cells in the final field, and recorded the numbers on an appropriate worksheet. When the counting was completed, parasite densities for all participants were calculated using an estimated average white cell count of 8000/μL for children aged more than five years. In addition, we used the white blood cells (WBCs) reference values established for children less than five years (9200/μL). The following formula was used for the calculation [30]:


$$\text{Parasites/}\mu \text{L blood}=\frac{\text{Number of parasites counted }\times \text{ Number of white blood cells/}\mu \text{L}}{\text{Number of white blood cells counted}}$$

**Study antigens:** the antigens consisted of recombinant Qβ displaying *Plasmodium falciparum* 3D7 strain sequence derived N-terminal part of MSP3 (QβMSP3) and UB05 (QβUB05) generated in our group as previously described [9].

**Determination of IgG antibody responses specific to QβUB05 and QβMSP3:** the plasma levels of antibodies specific to the malaria antigens QβUB05 and QβMSP3 were determined using an ELISA assay as previously described by our group [31]. Briefly, high binding ELISA plates were coated with 10^7^ particles/well of each recombinant phage and incubated overnight at 4°C. The following day, plates were washed 3x with Phosphate-Buffered Saline Tween (PBST) (PBS containing 0.05% Tween 20) and blocked with 3% BSA in PBS for one hour at 37 °C. Heat inactivated plasma samples were diluted in PBS at 1: 500 then 100 μl/well added in duplicate and incubated for two hours at 37 °C. The plates were washed four times with PBST after which the bound antibody was probed with the peroxidase-conjugated mouse anti-human IgG (Southern Biotech, Birmingham USA) diluted 1: 4000 in 1X PBS. The bound conjugate was detected using ABTS substrate and stop solution according to the manufacturer´s protocol (Southern Biotech, Birmingham USA). The colorimetric signal was measured at 405 nm using a multiscan FC microplate reader (Thermo Fisher Scientific, USA).

**Data analysis:** data was entered in Excel 2013 and analysis was done using Excel and Graphpad Prism Software version 6.1. Continuous variables from children´s characteristics and IgG antibody profiles were described as medians and Inter Quartile Ranges (IQR) and categorical variables were presented as percentages or proportions. Whereas the Student´s t-test was used to compare the means of two independent random samples (Bimodal compared to monomodal or MSP3 compared to UBO5), the Pearson correlation coefficient denoted by r, were used to measure the strength of a linear association between *Pf* parasite load and the IgG antibody responses specific to recombinant MSP3 and UB05. Moreover, all statistical tests were performed at a 95% confidence interval and the differences were considered as significant when the p-value was < 0.05. Data were presented in the form of tables and charts.

**Ethics approval and consent to participate:** this study received ethical approval from the Cameroon National Ethics Committee for Human Health Research (reference numbers 2015/03/561/CE /CNERSH/SP and 2018/01969/CE/CNERSH/SP) and the CIRCB institutional review board (protocol number 14-11). All participants provided written informed consent. Data were processed using specific identifiers for privacy and confidentiality purposes. Clinical data generated during the course of this study was provided free of charge to all participants.

**Availability of data and materials:** all data are fully available without restriction. Data are available from the CIRCB Institutional Data Access/Ethics Committee for researchers who meet the criteria for access to confidential data. All requests for data should be addressed to the general manager of CIRCB Prof. Alexis Ndjolo.

## Results

**Socio-demographic characteristics of the study population:** a total of 68 children were selected in Bikop; an area with bimodal rainfall characteristics and 66 children in Buea an area with monomodal rainfall features. The median age of children was 3.89 ± 0.78 years in Bikop (bimodal rainfall area) and 2.67 ± 1.57 years in Buea (monomodal rainfall area). The mean body temperature (36.42 ± 0.5) of healthy children living in bimodal rainfall areas was significantly lower (p<0.0001) than for their counterparts living in monomodal rainfall areas (37.09 ± 0.76) (Table 1).

**Table 1:** socio-demographic characteristics of the study population

Variables	Bikop (n=68)^a^	Buea (n=66)^a^
Age range: median (IQR)^b^	4 (3 - 4.75)	2.5 (2 - 4)
**Sex: n (%)^c^**		
Female	28 (41.18)	36 (54.55)
Male	40 (58.82)	30 (45.45)
Temperature (°C)^d^: mean ± SD^e^	36.42 ± 0.5	37.09 ± 0.76
Proportion of malaria positive cases: n (%)	33 (48.53)	19 (28.79)

a : number; ^b^: inter quartile ranges; ^c^: percentage; ^d^: degree celsius; ^e^: standard deviation

**Figure 1:**
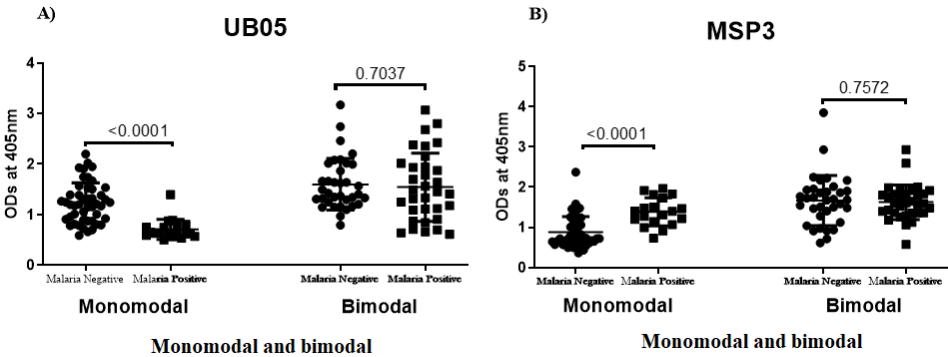
(A,B) comparison of the IgG antibody responses specific to QβUB05 and QβMSP3 in malaria negative and positive children

**IgG antibody responses specific to QβUB05 and QβMSP3 in malaria negative and positive children:** children living in bimodal and monomodal rainfall areas were screened for *Plasmodium falciparum* infection. In Figure 1 (A,B), data is shown for IgG antibody responses specific to either recombinant QβUB05 or QβMSP3 in plasma of children from both monomodal and bimodal rainfall areas. A comparison is made between malaria-infected and malaria-negative children. In Figure 1A, asymptomatic *Pf*-negative children show significantly higher (P<0.0001) IgG responses than *Pf*-positive children. With respect to MSP3 as shown in Figure 1B the converse was true as *Pf*-positive children showed significantly higher IgG responses (P<0.0001).

In monomodal rainfall areas, *Plasmodium falciparum* negative children showed significantly higher IgG response against QβUB05 (p<0.0001) when compared to their asymptomatic *Plasmodium falciparum-*infected counterparts (Figure 1A), significantly higher (p<0.0001) IgG reactivity to QβMSP3 was observed rather in asymptomatic *Plasmodium falciparum-*infected children in the same population (Figure 1B). However, the two vaccine candidates in bimodal rainfall areas did not show any significant difference with respect to IgG reactivity to samples from both *Plasmodium falciparum-*infected and negative children (Figure 1(A,B)).

**Figure 2:**
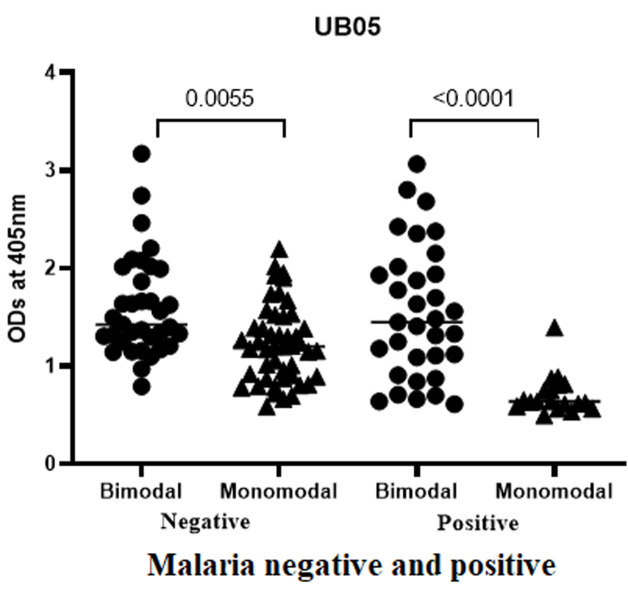
comparison of the IgG antibody responses specific to recombinant QβUB05 with respect to rainfall characteristics of the study areas

**IgG antibody response specific to QβUB05 in plasma from children of both bimodal and monomodal rainfall areas:** IgG antibody responses specific to recombinant QβUB05 were compared between children living in bimodal with monomodal rainfall areas. In Figure 2 data is shown comparing asymptomatic *Plasmodium falciparum-*infected and negative children from both rainfall areas. IgG antibody responses specific to QβUB05 were significantly higher in bimodal rainfall areas than in monomodal rainfall areas irrespective of children's *Pf* infection.

Generally, *Pf* transmission intensity is lower in monomodal rainfall areas relative to bimodal rainfall areas. Higher *Pf* transmission intensity implies that children living in bimodal rainfall areas suffer also intense exposure to *Pf* derived antigens including UBO5. Therefore as shown in Figure 2 IgG antibody responses specific to QβUB05 were significantly higher in bimodal than monomodal rainfall areas irrespective of children *Plasmodium falciparum* infection (p<0.0055 for positive and p<0.0001 for negative children) (Figure 2). Thus IgG antibody response was proportional to the intensity of the antigen challenging the children´s immune system. This was confirmed in the parasite load antibody correlation curves.

**Figure 3:**
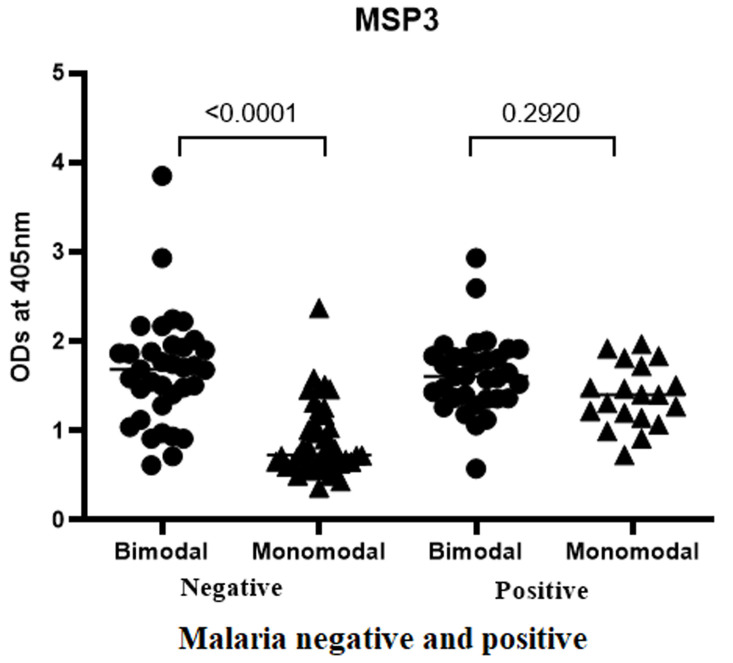
comparison of the IgG antibody responses specific to recombinant QβMSP3 with respect to rainfall characteristics of the study area

**IgG antibody response specific to QβMSP3 in plasma from children of both bimodal and monomodal rainfall areas:** IgG antibody responses specific to recombinant QβMSP3 were compared between children living in bimodal with monomodal rainfall areas. In Figure 3 data is shown comparing asymptomatic *Plasmodium falciparum-*infected and negative children from both rainfall areas. IgG antibody responses specific to QβMSP3 were significantly higher in bimodal rainfall areas than in monomodal rainfall areas only in *Plasmodium falciparum-*negative children.

Generally, *Pf* transmission intensity is higher in bimodal rainfall areas relative to monomodal rainfall areas. Thus, children living in bimodal rainfall areas suffer intense exposure to *Pf* derived antigens including MSP3. Therefore, as shown in Figure 2 IgG antibody responses specific to QβMSP3 were significantly higher in bimodal than monomodal rainfall areas only in *Plasmodium falciparum* negative children (P<0.0001) (Figure 3). In contrast, no significant difference was observed in asymptomatic *Plasmodium falciparum-*infected children (p=0.2920). Thus MSP3, probably due to high polymorphism is less sensitive in detecting changes in antigen load. Moreover, only the C-terminal region of MSP3 was engineered upon the surface of the recombinant phages.

**Figure 4:**
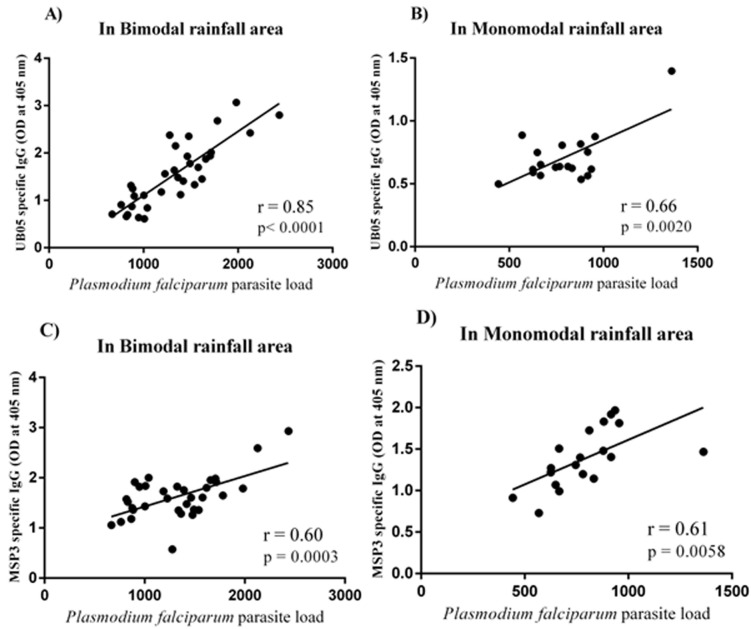
(A,B,C,D) correlation between the *Plasmodium falciparum* parasite load and the IgG antibody responses specific to QβUB05 and QβMSP3

**Correlation between the parasite load and the IgG reactivity to QβUB05 and QβMSP3:** the parasite load of asymptomatic *Plasmodium falciparum-*infected children living in bimodal and monomodal rainfall areas determined and correlated with the IgG antibody responses specific to QβUB05 and QβMSP3. In Figure 4A and Figure 4B data is shown for correlation between the malaria parasite load and the IgG antibody responses specific to QβUB05 from both rainfall areas. In Figure 4C and Figure 4D data is shown for correlation between the malaria parasite load and the IgG antibody responses specific to QβMSP3 from both rainfall areas.

The antibody response should be proportional to the intensity of exposure to the antigen. Data in Figure 4 (A,B,C,D) shows the correlation between the malaria parasite load and the IgG antibody responses specific to QβUB05 and QβMSP3. Significantly positive correlation was observed between the *Pf* load and the IgG antibody responses specific to QβUB05 ((r=0.85; p<0.0001 in bimodal rainfall area) (r=0.85; p<0.0001 in bimodal rainfall area) (r=0.66; p=0.0021 in monomodal rainfall area) (Figure 4 (A,B)) and QβMSP3 (r=0.60; p=0.0002 in bimodal rainfall area) (r=0.61; p=0.0053 in monomodal rainfall area) (Figure 4 (C,D)). It is worth noting that the correlation was stronger between the intensity of the antigen and the IgG antibody response specific to QβUB05 compared to the IgG antibody response specific to QβMSP3 irrespective of the rainfall conditions of the areas.

## Discussion

In this population-based cross-sectional study we profiled IgG responses specific to MSP3 and UB05 in plasma from asymptomatic *Plasmodium falciparum-*infected and negative children living in two localities of Cameroon differing in rainfall characteristics. In asymptomatic *P. falciparum* negative children IgG antibody responses specific to UBO5 in monomodal rainfall areas were surprisingly significantly higher (p<0.0001) than in asymptomatic *P. falciparum-*infected children. Ongoing *P. falciparum* infection is expected to increase targeted antigen expression and as such drives the expansion of memory B cells leading to increased UBO5 specific IgG antibody titres. Strangely this was not the case when *P. falciparum* negative and positive children were compared in the monomodal rainfall areas. This is probably due to previously induced long-lived UB05-specific memory B cells which are essential in maintaining immunity against malaria in the negative participants.

Generally, in malaria-endemic areas submicroscopic parasitaemia could challenge the immune system continuously with antigens that drive the expansion and maturation of long-lived memory cells which could be highly potent in parasite clearance [31,32]. The fact that UBO5-specific antibody responses in monomodal rainfall areas were even higher than in asymptomatic *Plasmodium falciparum-*infected children probably suggests that such memory cells specific to UBO5 are probably highly sensitive to their stimulating antigen and expanded accordingly. In contrast, MSP3-specific IgG antibody responses in the monomodal areas were likely more reliant upon an ongoing high parasitaemia as *Plasmodium falciparum-*infected children showed significantly higher (p<0.0001) IgG antibody responses than their negative counterparts. In bimodal rainfall areas where there is proportionately also high *P. falciparum* transmission intensity challenging the immune system with high amounts of antigens; UBO5 specific IgG antibody responses were significantly higher irrespective of malaria infection (P<0.0055 *P. falciparum* negative or P<0.0001 for *P. falciparum* positive children). This clearly shows that UBO5-specific B cells produced antibodies proportionately to the circulating amount of antigens. This was in contrast with MSP3-specific IgG antibody responses which were significantly higher only in *P. falciparum* negative children (P<0.0001).

During *P. falciparum* infection no significant difference is observed between bimodal and monomodal rainfall areas with respect to MSP3-specific IgG antibody responses (P=0.2920). Several *Plasmodium spp* derived blood-stage antigens are known to play essential roles in naturally acquired immunity to malaria which is vital in reducing the multiplication of the parasite and thus preventing infection and clinical disease in long-term residents of endemic areas [7,8]. There is an accumulation of evidence that effective anti-malaria vaccines targeting blood-stage parasites must be an essential component of future malaria vaccines [33]. This is mainly because a number of studies have demonstrated an association between high concentrations of IgG-specific to asexual stage antigens and a substantial reduction of parasitemia and clinical symptoms [34]. There are several variants of MSP which have been shown to typically induce high titers of antibodies in people living in malaria-endemic regions. This makes the MSP3 an unreliable candidate for tracking malaria immunity in endemic regions. UBO5 is a more suitable candidate for monitoring naturally acquired immunity against malaria, especially across areas differing in malaria transmission intensity.

Our results indicate that the antibody response to these potential malaria vaccine candidates is induced early in children and could vary depending upon the climatic conditions driving parasite transmission intensity [35-38]. Therefore, in areas with bimodal rainfall and two annual peaks of exposure to *Plasmodium falciparum* infection, children are more exposed and thereby more likely to develop a higher immune response against MSP3 and UB05 compared to children from Buea with monomodal rainfall and one annual peak of exposure to *Plasmodium falciparum* infection. Our data demonstrates that UB05-specific antibody responses could be more reliable for tracking partial naturally acquired immunity to malaria irrespective of the climatic conditions and malaria transmission intensity. In addition, UB05 is also a *Plasmodium falciparum* immunodominant antigen, which had previously been demonstrated to reliably predict semi-immunity to malaria in adults living in a monomodal area of Cameroon [9,13]. Our work extends these findings not only to children but equally highlights its importance as a component for future malaria vaccine candidates applicable also in binomial rainfall areas.

## Conclusion

Overall, the IgG antibody responses specific to both antigens are relatively heterogeneous being certainly influenced by a number of factors including the nature of the antigen, regional rainfall conditions, and malaria parasite load. Nevertheless, antibody responses specific to UBO5 demonstrated a clear indication of naturally acquired protective immunity against *Pf* infection and increased proportionately with antigen load. Thus antibody responses specific to UB05 were more reliable in predicting partial naturally acquired immunity to malaria. UBO5 therefore should be considered as a necessary component of future malaria vaccine candidates. On the other hand, IgG to both MSP3 and UB05 was significantly higher in children living in bimodal as compared to those living in monomodal rainfall areas. Thus the differential rainfall characteristics of these two rainfall areas could modulate malaria transmission intensity which in effect results in differing IgG antibody reactivity to potential malaria vaccine candidates. In other to avoid choosing an unsuitable universal malaria vaccine candidate for clinical trials it is necessary to integrate climatic conditions during malaria vaccine candidate optimization.

### What is known about this topic

- *Immunization of albino, laboratory-bred strain of the house mouse (BALB/c mice) with a recombinant chimera of UB05 and UB09 (UB05-09), blocked parasitemia;*
- *Antibodies specific to Plasmodium falciparum MSP3 have the ability to control P. falciparum parasite density by triggering the blood monocytes through antibody-dependent cellular inhibition (ADCI);*
- *IgG and subclass-specific responses to Plasmodium falciparum MSP vary considerably in children from the different bioecological strata in Cameroon*.

### What this study adds

- *We show that varying ecological conditions coupled with pf transmission intensities result to differential IgG antibody responses specific to MSP3 and UBO5;*
- *IgG to both MSP3 and UB05 is significantly higher in children living in bimodal rainfall areas relative to those living in monomodal rainfall area;*
- *Antibody responses specific to UB05 are more reliable in predicting partial immunity to malaria in children irrespective of bimodal or monomodal rainfall area. Proving its suitability for further optimization as a global malaria vaccine candidate*.
